# NSUN6 Maintains BMPER Stability in an m5C‐Dependent Manner to Suppress Cell Proliferation and Migration in Hepatocellular Carcinoma

**DOI:** 10.1111/boc.70023

**Published:** 2025-06-30

**Authors:** Chunlin Liu, Yumin Wu, Ying Wu, Weizhi Luo, Yuefei Hong, Leichang Jiang, Senrui Wang, Duanming Du

**Affiliations:** ^1^ Department of Interventional Therapy Shenzhen Second People's Hospital (First Affiliated Hospital of Shenzhen University) Shenzhen China

**Keywords:** BMPER, hepatocellular carcinoma, migration, m5C, NSUN6, proliferation

## Abstract

**Background:**

The expression of Nop2/Sun domain family member 6 (NSUN6), an RNA m5C methyltransferase, is correlated with the prognosis of various cancers. However, its role in the progression of hepatocellular carcinoma (HCC) remains elusive.

**Methods:**

The expression of NSUN6 was analyzed using the TCGA‐HCC cohort, as well as through quantitative real‐time reverse transcription polymerase chain reaction (qRT‐PCR) and western blotting in tumor tissues from HCC patients and various HCC cell lines. Moreover, its biological functions were detected using cell counting kit 8 (CCK8), colony formation, 5‐ethynyl‐2′‐deoxyuridine (EdU), wound healing, and transwell invasion assays in vitro, as well as in an HCC patient‐derived xenograft (PDX) mouse model in vivo. The molecular mechanism underlying NSUN6 was explored using methylated RNA immunoprecipitation sequencing (MeRIP‐seq), double luciferase reporter gene assay, actinomycin‐D assay, and rescue experiments in SNU449 cell lines.

**Results:**

The expression level of NSUN6 was significantly decreased in the TCGA‐HCC cohort, tumor tissues of HCC patients and HCC cell lines. NSUN6 overexpression markedly inhibited the proliferative and migratory abilities of HCC cells in the PDX mouse model. Additionally, BMPER was identified as a downstream target of NSUN6, while NSUN6 could stabilize BMPER expression in an m5C‐dependent manner. Finally, BMPER knockdown reversed the positive effects of NSUN6 in suppressing HCC progression.

**Conclusion:**

This study elucidated the inhibitory effect of NSUN6 overexpression in HCC development, with BMPER identified as a downstream target of NSUN6. NSUN6 regulates BMPER expression in an m5C‐dependent manner, thereby influencing HCC progression. Overall, these results suggest that the NSUN6/BMPER axis may serve as a potential therapeutic target for HCC.

## Graphical abstract

In hepatocellular carcinoma (HCC), the methyltransferase NSUN6 catalyzes m⁵C modification of BMPER mRNA, enhancing its stability and elevating BMPER protein expression. This NSUN6‐BMPER axis suppresses HCC progression by inhibiting tumor cell proliferation/migration while correlating with improved patient prognosis, establishing RNA epigenetic regulation as a key inhibitory mechanism in liver cancer.

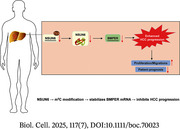

## 1 Introduction

As is well documented, hepatocellular carcinoma (HCC) is one of the most prevalent and deadly forms of primary liver cancer, accounting for 90% of cases (Forner et al. 2018). In East Asia and Africa, HCC has the highest incidence and mortality rates and is predicted to be the third leading cause of cancer‐related death by 2030 (Llovet et al. 2021). The high incidence and poor prognosis of HCC highlights its relevance as a critical area of cancer research. While knowledge about HCC is expanding, the mechanisms underlying its occurrence and development remain largely unknown, highlighting the necessity for a more thorough examination of its molecular mechanisms to explore novel therapeutic targets.

RNA post‐transcriptional modifications have emerged as crucial regulators of gene expression, affecting a wide range of diseases by influencing miscellaneous cellular processes such as RNA stability, localization, and translation (Barbieri and Kouzarides 2020, Nachtergaele and He 2017). Among these modifications, 5‐methylcytosine (m5C) has garnered extensive attention, given that it affects RNA stability in various cancer types, such as bladder cancer (Chen et al. 2019), esophageal squamous cell carcinoma (Su et al. 2021), non‐small‐cell lung cancer (Wang et al. 2023), and retinoblastoma (Zuo et al. 2023). Notably, the m5C modification involves a series of regulators, including m5C methyltransferases and demethylases (Chen et al. 2021). Nop2/Sun domain family member 6 (NSUN6) is a conserved RNA m5C methyltransferase that has been identified as a key enzyme catalyzing m5C modification on RNA by binding to three prime untranslated regions (3′‐UTRs) at the consensus sequence motif CTCCA, located in the loops of hairpin structures (Selmi et al. 2021). Prior research has linked NSUN6 to various cancers (Lu et al. 2024). For example, NSUN6 has been reported to mediate the m5C modification of NDRG1 mRNA and promote radioresistance in cervical cancer (Yu et al. 2024). In another study, NSUN6‐mediated m5C deposition regulated transcriptional pausing by promoting the accumulation of NELFB and general transcription factor complexes, which in turn influenced the response of glioblastoma to temozolomide (Awah et al. 2021). Based on the aforementioned studies, the dysregulation of m5C methylation and NSUN6 activity has been implicated in cancer progression, indicating that NSUN6 may serve as a potential target for cancer therapy. Nevertheless, the roles of m5C modifications and NSUN6 in HCC, along with their mechanisms of action, remain to be elucidated.

In the present study, NSUN6 was lowly expressed in HCC and associated with poor diagnosis, while NSUN6 overexpression suppressed cell proliferation, migration, and tumor growth. Mechanistically, NSUN6 exerted anti‐tumorigenic effects by maintaining BMPER stability through m5C modification. Thus, this study elucidated that the NSUN6‐m5C‐BMPER axis modulates HCC cell proliferation, positioning NSUN6 as a candidate therapeutic target for HCC.

## 2 Materials and Methods

### 2.1 Online Database Analysis

NSUN6 expression was analyzed using data from the Cancer Genome Atlas (TCGA) database (https://xenabrowser.net/datapages/), which comprised 160 peritumoral tissues and 369 hepatocellular carcinoma tissues.

Overall survival was determined using the Kaplan–Meier model (http://kmplot.com/analysis/).

The potential downstream m5C target genes of NSUN6 were predicted using multiple online databases, including RM2Target (http://rm2target.canceromics.org/#/home), GEO database (https://www.ncbi.nlm.nih.gov/geo/) with accession codes GSE202069 and GSE214846, and TCGA (https://www.cancer.gov/ccg/research/genome‐sequencing/tcga).

### 2.2 Cell Culture and Transfection

Human hepatic stellate cell lines (LX‐2) and human hepatocellular carcinoma cell lines (Hep3B, HCCLM3, SNU449) were sourced from Jennio Biotech (Guangzhou, China). The cells were cultured in a 10% DMEM complete medium supplemented with 1% penicillin/streptomycin (Life Technologies, Carlsbad, CA, USA) at 37°C in an atmosphere containing 5% CO_2_. The HCC cell lines (Hep3B, HCCLM3, and SNU449) were selected based on their molecular subtypes and clinical relevance: Hep3B represents TP53‐deficient HCC, widely used for studying proliferation and apoptosis mechanisms (Yu et al. 2023). HCCLM3 is a high‐metastatic‐potency model suitable for migration and invasion assays (Yuan et al. 2022). SNU449 retains wild‐type β‐catenin, reflecting the Wnt signaling‐activated subtype (Zhang et al. 2024). This selection ensures coverage of diverse HCC molecular profiles and enhances translational relevance.

The lentiviruses for NSUN6 overexpression and BMPER knockdown were procured from Genechem (Shanghai, China). NSUN6 overexpression lentivirus was named NSUN6‐OE, whereas the control lentivirus was termed Ctrl. BMPER knockdown lentivirus was designated as sh‐BMPER, and a control lentivirus was referred to as sh‐NC. SNU449 cells were infected with the aforementioned lentiviruses in the presence of 8 mg/mL polybrene for 48 h. The stably transfected cells were selected by incubating in DMEM media containing 1 g/mL puromycin for 2 weeks. The transduction efficiency was evaluated via quantitative real‐time reverse transcription‐polymerase chain reaction (qRT‐PCR).

### 2.3 Quantitative Real‐Time Reverse Transcription Polymerase Chain Reaction (qRT‐PCR)

Total RNA was extracted using the standard TRIzol (Invitrogen, Carlsbad, CA, USA) and reverse transcribed into cDNA using the reverse transcription kit (Takara, Dalian, China). Quantitative real‐time reverse transcription polymerase chain reaction (qRT‐PCR) was performed using SYBR Green Mix (Takara, China) on an Applied Biosystem Prism 7500 Fast Sequence Detection System (Applied Biosystems, Foster City, CA, USA). Relative expression was calculated using the 2^–ΔΔCt^ method. The primer sequences used for amplification are listed in Table 1.

**TABLE 1:** The sequences of primers and oligonucleotides used in this study.

Gene	Sequences:5′‐3′
NSUN6‐F	5′ ATGCACCCTGTAGTGGAATGG 3′
NSUN6‐R	5′ ACCTGTTCTTCATTTTCGGCC 3′
BMPER‐F	5′ ATCGACCTGGATGGCTACCTC 3′
BMPER‐R	5′ GAAGTTTCCATCTCCACCAATTA 3′
GAPDH‐F	5′ GGATTTGGTCGTATTGGG 3′
GAPDH‐R	5′ GGAAGATGGTGATGGGAT 3′

### 2.4 Western Blot Analysis

Cells or tissues were lysed using RIPA buffer (Beyotime Biotechnology, Shanghai, China) to extract the total protein, and the total protein was detected by the BCA Protein Assay Kit (Beyotime Biotechnology). Equal amounts of protein samples were separated by 10% SDS‐PAGE and then transferred onto the PVDF membrane. Next, the membrane was blocked with 5% nonfat milk at 25°C for 2 h and incubated with primary antibodies against NSUN6 (1:1,000; Affinity Biosciences, Cincinnati, OH, USA), BMPER (1:300; Thermo Fisher Scientific, Inc., Waltham, MA, USA), and GAPDH (1:3000, Affinity Biosciences) at 4°C overnight. Afterward, it was incubated with horseradish peroxidase (HRP)‐conjugated secondary antibodies (1:3000; Affinity Biosciences) at room temperature for 2 h. Protein expression was visualized using an enhanced chemiluminescence (ECL) detection system.

### 2.5 CCK‐8 Proliferation Assay

SNU449 and HCCLM3 cells in each group were seeded in 96‐well plates. Then, 10 µL of CCK‐8 reagent (Sigma–Aldrich, St. Louis, MO, USA) was added to each well at 0, 24, 48, and 72 h. Absorbance was measured at 450 nm using a microplate reader (Fisher Scientific, Waltham, MA, USA).

### 2.6 Colony Formation Assay

Cells from each group were seeded into 6‐well plates, and the culture medium was replaced every 48 h and discarded after 2 weeks of culture. Cells were then fixed with 4% paraformaldehyde for 30 min after washing in PBS and then stained with 4% crystal violet. The number of clones was counted under a microscope (Zeiss Axio Vert.A1, Jena, Germany).

### 2.7 5‐Ethynyl‐2′‐Deoxyuridine (EdU) Assay

Cells were seeded in a 24‐well plate for 48 h and then incubated with 50 µM 5‐ethynyl‐2′‐deoxyuridine (EdU) (Beyotime Biotechnology, Shanghai, China) for 2 h at 37°C. After fixing with 4% paraformaldehyde, the cells were stained with 4′,6‐diamidino‐2‐phenylindole (Beyotime Biotechnology, Shanghai, China) for 30 min. The proportion of EdU‐positive cells was determined using fluorescence microscopy (Olympus, Tokyo, Japan).

### 2.8 Wound‐Healing Assay

The cells were seeded into six‐well plates and cultured to 90% confluence (>90%). Wounds were created in the center of each cell using the tip of a sterile 200 µL plastic pipette. Cellular debris was washed away with PBS, following which the cells were incubated for 24 h. The wound was imaged at 0 and 24 h under a microscope (Olympus, Tokyo, Japan).

### 2.9 Transwell Assay

Cells were seeded into the upper Transwell chambers with a serum‐free medium at a density of 2 × 10^4^ cells/mL. Meanwhile, a 10% DMEM medium was added to the lower compartment. After incubating at 37°C for 24 h, migrated cells in the lower chamber were fixed with paraformaldehyde and stained with 0.1% crystal violet, followed by counting using a light microscope (Olympus).

### 2.10 Patient‐Derived Xenografts (PDX) Mouse Model

All animal experiments were performed in accordance with the ARRIVE Guidelines and approved by the Animal Ethics Committee of Guangzhou Jennio Biotech Co. Ltd. (Approval No. JENNIO‐IACUC‐2023‐A067). A total of 12 NCG male mice (6 weeks old) were obtained from the Laboratory Animal Center of Southern Medical University and provided with ad libitum access to food and water and housed under controlled conditions (Temperature 22.2°C, air humidity 40%–70%) with a 12 h:12 h light/dark cycle. To construct the PDX model, fresh tumor tissues were collected from liver cancer patients and subsequently sliced into appropriately sized small sections. These pieces were then implanted into the right subcutaneous area of mice. Upon the tumor volume reaching 1000 mm^3^, the mice were euthanized. Tumor tissues were collected, sliced into small segments (2 mm^3^), and implanted into the right subcutaneous area of mice. When the tumor size reached approximately 100 mm^3^, the mice were randomly assigned to two groups (*n* = 6): negative control group and NSUN6 overexpression group. In the negative control group, mice were injected with 1 × 10^9^ PFU/mL control lentivirus into the tail vein. In the NSUN6 overexpression group, mice were injected with 1 × 10^9^ PFU/mL NSUN6 overexpression lentivirus into the tail vein. The tumor size was measured every 4 days and calculated according to the following formula: V = (length × width^2^)/2. Tumor tissues were harvested for subsequent analysis.

### 2.11 Hematoxylin and Eosin (H&E) Staining

Mouse hepatic specimens were fixed with 4% paraformaldehyde and paraffin‐embedded, followed by sectioning into 5 µm slices. After dewaxing and rehydration, the slices were stained using hematoxylin and eosin (H&E) solution (Sigma–Aldrich, St. Louis, MO, USA) and examined under an Olympus light microscope (Japan).

### 2.12 Immunohistochemistry (IHC) Staining

Paraffin‑embedded tissue specimens were sectioned into 5 µm slices and then dewaxed and rehydrated using graded ethanol, followed by incubation with primary antibodies against PCNA (1:100, Affinity Biosciences) at 4°C overnight. The sections were washed with PBST and subsequently incubated with horseradish peroxidase (HRP)‐conjugated secondary antibodies (1:200; Affinity Biosciences) at room temperature for 1 h. Protein expression was detected using the DAB Kit (Beyotime Biotechnology, Shanghai, China). The nuclei were counterstained using hematoxylin (Sigma–Aldrich, St. Louis, MO, USA). Images were captured under a light microscope (Olympus Corp.) using an Olympus DP70 digital camera.

### 2.13 Methylated RNA Immunoprecipitation (MeRIP) Assay

Total RNAs were extracted from cells using Trizol reagent and treated with an RNase‐free DNase set (Qiagen, Germany). After immunoprecipitation with an anti‐m5C antibody (ProteinTech Group, Chicago, IL, USA), the chemically fragmented RNAs (∼100 nucleotides) were washed with RIP buffer three times. RNAs were eluted from the beads following incubation with elution buffer (Sigma–Aldrich) for 1 h at 4°C. After ethanol precipitation, the input and eluted RNAs were collected for the ensuing experiments.

### 2.14 Dual‐Luciferase Reporter Assay

The wild‐type (wt) and mutant (mut) BMPER promoter binding sites were subcloned into psiCHECK‐2 plasmids (Promega, Madison, WI, USA) and co‐transfected with lentivirus overexpressing NSUN6 (Genechem) into SNU449 and HCCLM3 cells. After 48 h, luciferase activity was measured using a dual‐luciferase assay kit (Promega, Madison, WI, USA).

### 2.15 Actinomycin D (Act D) Assay

SNU449 cells were exposed to 2 µg/mL actinomycin D (Act D, Sigma–Aldrich). Total RNAs were extracted at 0, 2, 4, 6, and 8 h. The relative mRNA expression of BMPER was analyzed using qRT‐PCR.

### 2.16 Statistical Analysis

Statistical analysis was performed using SPSS 21.0 (SPSS Inc., Chicago, IL, USA). Data were presented as mean ± standard deviation (SD), and differences between groups were analyzed using a two‐tailed Student *t*‐test, one‐way ANOVA, or two‐way ANOVA. A *p* value <0.05 was considered statistically significant.

## 3 Results

### 3.1 NSUN6 is Lowly Expressed in Hepatocellular Carcinoma Tissues and Cell Lines

**FIGURE 1:**
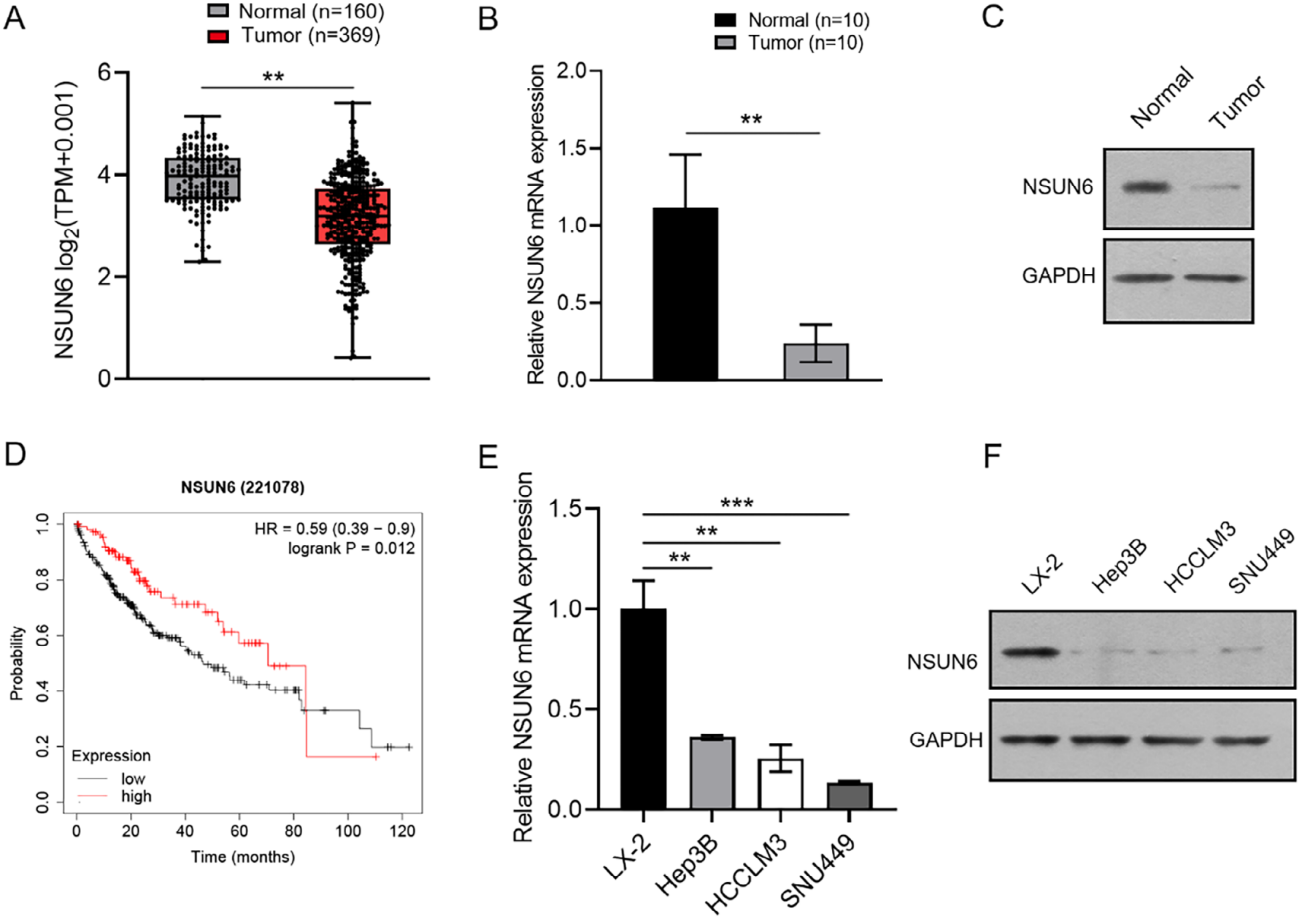
NSUN6 is lowly expressed in hepatocellular carcinoma tissues and cell lines. (A) The TCGA database was used to analyze NSUN6 expression in HCC samples. (B and C) qRT‐PCR and western blot assays were conducted to evaluate NSUN6 expression in HCC clinical samples. (D) Kaplan–Meier curves were plotted to assess the correlation between NSUN6 expressions and overall survival rates among HCC patients. (E and F) NSUN6 expression in HCC cell lines was detected using qRT‐PCR and western blot analysis. All experiments were performed in triplicate. Values are presented as the mean ± SD of three independent experiments. *p < 0.05, **p < 0.01, ***p < 0.001.

TCGA analysis revealed that NSUN6 expression was significantly lower in HCC tissues compared to peritumoral tissues (Figure 1A). Likewise, qRT‐PCR and western blotting assays demonstrated that NSUN6 expression was markedly lower in tumor tissues compared with peritumoral tissues from HCC patients (Figure 1B,C). Kaplan–Meier survival curves illustrated that low NSUN6 expression (*n* = 245) predicted poorer overall survival probability in HCC patients compared to high NSUN6 expression (*n* = 110), highlighting the association between low NSUN6 expression and poor prognosis (Figure 1D). Furthermore, western blotting and qRT‐PCR assays unveiled that NSUN6 expression levels were significantly lower in HCC cell lines (Hep3B, HCCLM3, and SNU449) compared to the human normal hepatic cell line (LX‐2) (Figure 1E,F), supporting the trend of NSUN6 downregulation in HCC and providing a basis for further functional studies.

### 3.2 Ectopic Over‐Expression of NSUN6 Inhibits HCC Cell Proliferation and Migration

**FIGURE 2:**
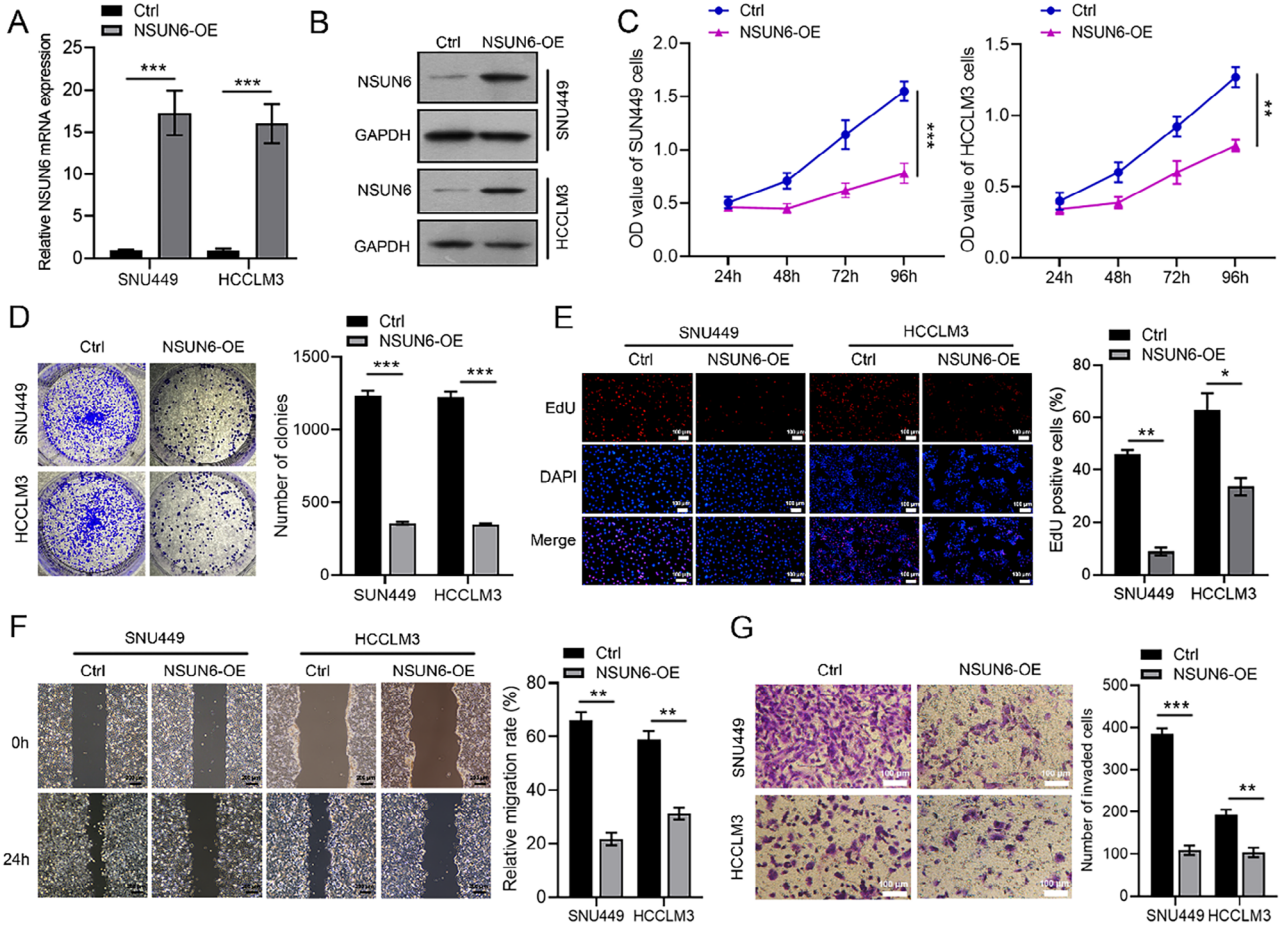
Effects of NSUN6 overexpression on HCC proliferation and migration. (A and B) qRT‐PCR and western blot analysis were conducted to confirm the successful construction of NSUN6 overexpression in SNU449 and HCCLM3 cell lines. (C–G) CCK‐8 (C), colony formation (D), EdU (E), wound healing (F), and Transwell invasion (G) assays were performed to explore the effects of NSUN6 overexpression on the proliferative and migratory abilities of HCC cells. All experiments were performed in triplicate. Values are expressed as the mean ± SD of three independent experiments. *p < 0.05, **p < 0.01, ***p < 0.001.

SNU449 and HCCLM3 cells were transfected with NSUN6‐overexpressing lentivirus to investigate the functional impact of NSUN6. qRT‐PCR and western blot analysis confirmed the successful construction of NSUN6 overexpression (Figure 2A,B). Subsequent functional assays, including CCK‐8, colony formation, EdU, wound healing, and Transwell invasion assays, revealed that NSUN6 overexpression substantially reduced the proliferative and migratory abilities of HCC cells (Figure 2C–G). Taken together, these results suggested that NSUN6 up‐regulation acts as a tumor suppressor by inhibiting the proliferation and migration of HCC cells.

### 3.3 NSUN6 Overexpression Inhibits the Progression of Hepatocellular Carcinoma in an In Vivo PDX Model

**FIGURE 3:**
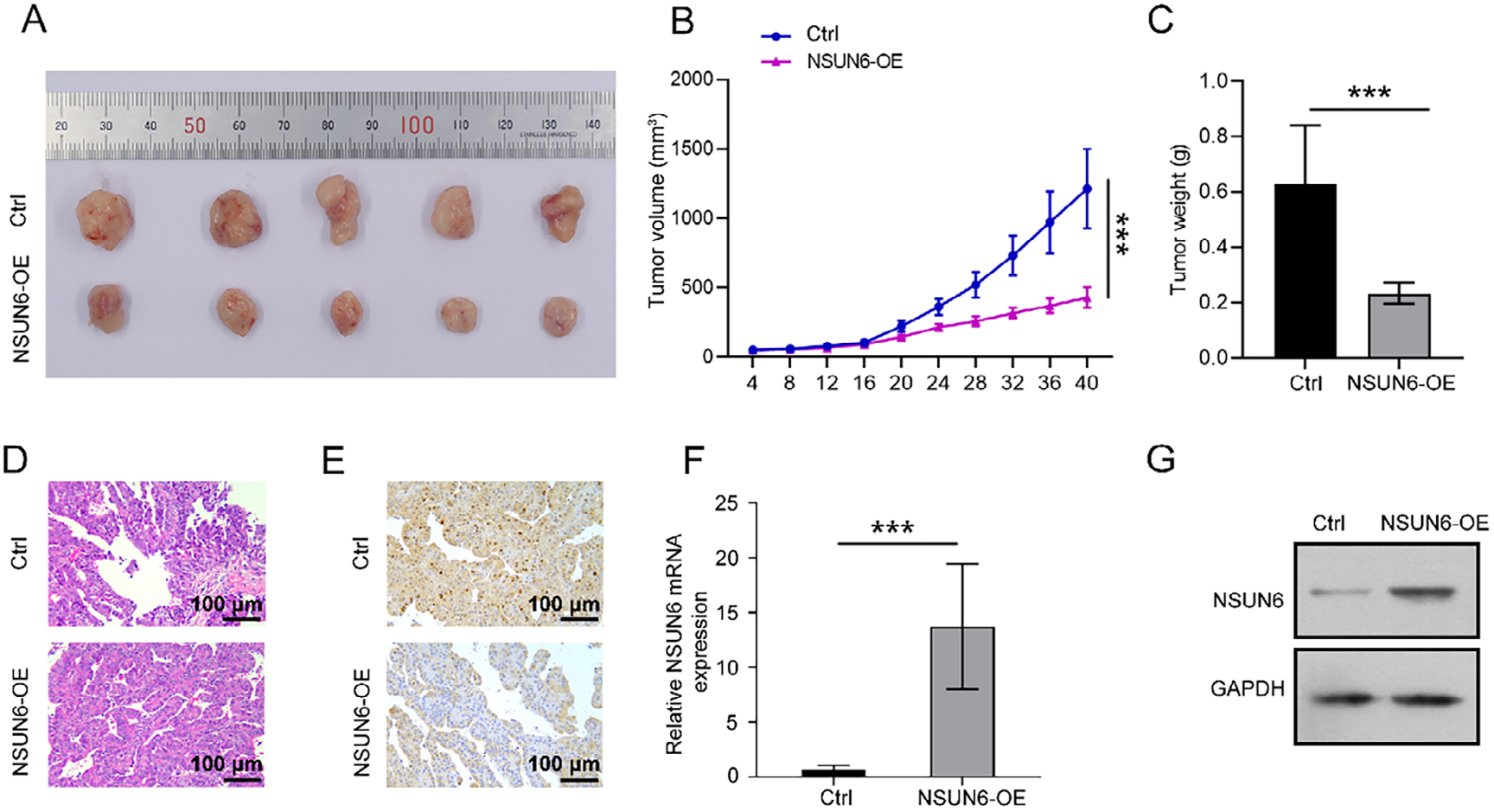
Impact of NSUN6 overexpression on tumor growth in a hepatocellular carcinoma PDX model. (A–C) Tumor growth, volume, and weight in PDX mice across each group. (D) H&E staining of tumor tissues across groups. (E) IHC staining for PCNA across each group. (F and G) qRT‐PCR and western blot analyses were conducted to determine NSUN6 expression in PDX tissues and peritumoral tissues. All experiments were performed in triplicate. Values are presented as the mean ± SD of three independent experiments. *p < 0.05, **p < 0.01, ***p < 0.001.

Furthermore, the effect of NSUN6 overexpression on tumor growth was assessed in the PDX mouse model. The results showed that the tumor volume and weight were significantly lower in the NSUN6 overexpression group compared to the control group (Figure 3A–C). H&E staining indicated enhanced proliferation of HCC cells in mice in the control group, while NSUN6 overexpression resulted in varying degrees of necrosis in cancer cells (Figure 3D). At the same time, IHC staining revealed lower PCNA‐positive cells in the NSUN6 overexpression group (Figure 3E). Meanwhile, qRT‐PCR and western blot analysis confirmed the higher expression level of NSUN6 in PDX tissues following lentivirus delivery, compared with its expression in peritumoral tissues (Figure 3F,G). These findings conjointly suggested that NSUN6 overexpression effectively suppressed tumor growth in vivo.

### 3.4 NSUN6 Maintains BMPER Stability in m5C Manner

**FIGURE 4:**
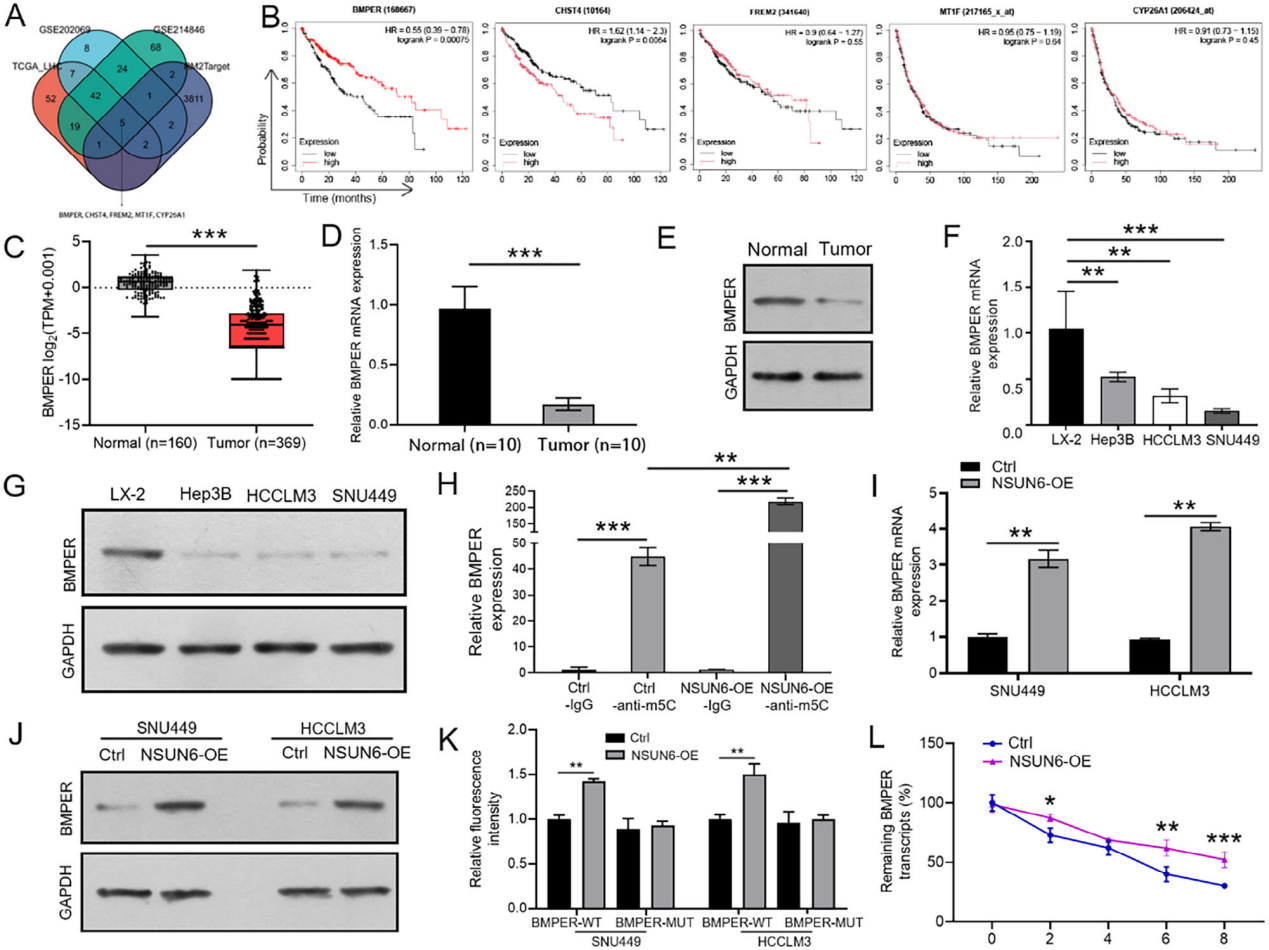
NSUN6 stabilizes BMPER mRNA through an m5C‐dependent mechanism. (A) Bioinformatics analysis was performed to identify five potential m5C target genes of NSUN6. (B) BMPER expression was correlated with a poor prognosis in HCC patients. (C) BMPER expression in HCC tissues was explored in the GEPIA database. (D–G) qRT‐PCR and western blot analyses were conducted to determine BMPER expression in clinical samples (D and E) and HCC cell lines (F and G). (H) m5C levels were detected using the m5C‐meRIP assay. (I and J) BMPER expression in NSUN6‐overexpressed SNU449 and HCCLM3 cells was determined using qRT‐PCR and western blot analyses. (K) The binding site between PKD and Neurod2 was confirmed by the dual‐luciferase reporter assay. (I) BMPER RNA stability was assessed using the Act D assay. All experiments were performed in triplicate. Values are presented as the mean ± SD of three independent experiments. *p < 0.05, **p < 0.01, ***p < 0.001.

The bioinformatics database RM2Target was used to identify the WER‐m5C targets of NSUN6 and cross‐reference targets with differentially expressed genes (DEGs) from GSE202069 (log2FC ≤ –4), GSE214846 (log2FC ≤ –4), and TCGA (log2FC ≤ –4) datasets to identify potential downstream m5C target genes of NSUN6, Finally, five candidate genes were identified, namely BMPER, CHST4, FREM2, MT1F, and CYP26A1 (Figure 4A). Among them, only BMPER was significantly associated with a poor prognosis in HCC patients (Figure 4B). Next, the GEPIA database was analyzed, and the results corroborated the decreased expression level of BMPER in HCC tissues (Figure 4C), which was further validated by qRT‐PCR and western blot analysis of clinical samples (Figure 4D,E) and different cell lines (Figure 4F,G). Subsequently, the m^5^C‐meRIP assay demonstrated that NSUN6 overexpression increased the m5C levels of BMPER (Figure 4H). Additionally, BMPER expression was upregulated in NSUN6‐overexpressed SNU449 and HCCLM3 cells (Figure 4I,J). Importantly, the dual‐luciferase reporter assay indicated that NSUN6 overexpression significantly increased the luciferase activities of BMPER‐Wt rather than that of BMPER‐Mut in SNU449 and HCCLM3 cell lines (Figure 4K). Furthermore, the Act D assay uncovered a longer half‐life of BMPER mRNA in the NSUN6‐OE group compared to the control group (Figure 4L). These results collectively suggested that NSUN6 overexpression enhanced BMPER mRNA stability in a m5C‐dependent manner.

### 3.5 BMPER Knockdown Reverses the Inhibitory Effect of NSUN6 Overexpression on HCC Cell Proliferation and Migration

**FIGURE 5:**
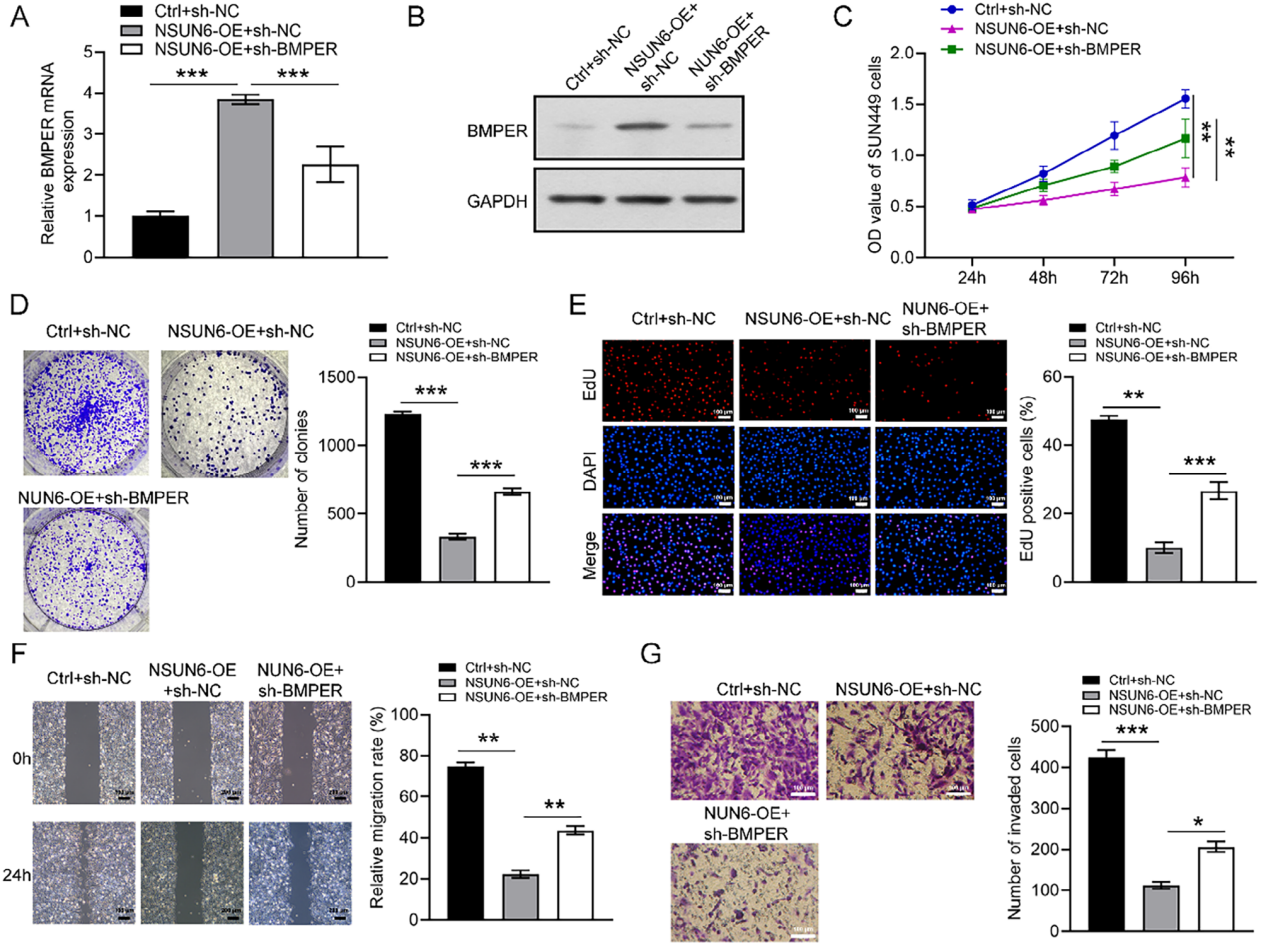
BMPER knockdown restored the inhibition of NSUN6 overexpression on the proliferation and migration of HCC cells. (A and B) The co‐transfection efficiency of oe‐NSUN6 and si‐BMPER in SNU449 cells was validated by qRT‐PCR and western blot analysis. (C–G) CCK‐8, colony formation, EdU, wound healing, and Transwell invasion assays were performed to examine the effect of BMPER knockdown on the proliferative and migratory capabilities of HCC cells. All experiments were performed in triplicate. Values are expressed as the mean ± SD of three independent experiments. *p < 0.05, **p < 0.01, ***p < 0.001.

BMPER knockdown lentivirus was transduced into SNU449 cells to further explore the underlying mechanism. The transfection efficiency was verified by qRT‐PCR and western blotting (Figure 5A,B). In cytological investigation, the proliferative and migratory capabilities of cells were effectively inhibited by NSUN6 overexpression, which was significantly reversed following BMPER knockdown (Figure 5C–G). These findings highlighted the pivotal role of BMPER in HCC cell proliferation and migration as a downstream effector of NSUN6.

## 4 Discussion

Hepatocellular carcinoma poses a global health challenge due to its high incidence, with both morbidity and mortality rates on the rise (Forner et al. 2018). Despite recent advancements, there remains an urgent need to identify new biomarkers for expanding our understanding of HCC and its management.

Aberrant m5C modifications in RNA have been linked to tumorigenesis and immune infiltration in various cancers, positioning them as novel targets for cancer therapy (Li et al. 2022). Recently, prognostic models utilizing m5C‐associated genes have been developed for HCC, providing new perspectives on the significance of this RNA modification in cancer progression (Feng et al. 2022). NSUN6, an m5C RNA methyltransferase, has recently garnered widespread attention for its role in cancer progression (Yang et al. 2021, Cui et al. 2024). Yang et al. reported that the expression level of NSUN6 was decreased in pancreatic cancer and could suppress tumorigenesis by modulating cell proliferation (Yang et al. 2021) Similarly, Lu et al. noted that NSUN6 expression was reduced in lung cancer cells and that NSUN6 could regulate NM23‐H1 expression by modifying the 3′‐UTR of NM23‐H1 mRNA through m5C and inhibit lung cancer cell proliferation, migration, and EMT (Lu et al. 2024). Herein, the expression level of NSUN6 was decreased in the TCGA‐HCC cohort, which was further demonstrated by qRT‐PCR and western blot analysis of HCC clinical samples and cell lines. Additionally, Kaplan–Meier analysis displayed that low expression of NSUN6 predicted a poor prognosis in HCC patients. According to earlier studies, high NSUN6 expression confers survival benefits in some cancer types (Awah et al. 2021), indicating its potential role as a tumor suppressor. Consistent with the findings of previous studies, both cytological and PDX‐model assays revealed that NSUN6 overexpression significantly impaired the proliferative and migratory abilities of HCC cells in this study, suggesting that enforced NSUN6 expression may represent a promising tumor suppressor in HCC.

Bone morphogenetic protein‐binding endothelial regulator (BMPER) is a secreted BMP‐binding glycoprotein (Heinke et al. 2008) first discovered in a screening process aimed at identifying proteins that are expressed differentially in embryonic endothelial precursor cells (Moser et al. 2003). It has been extensively studied in the field of vascular biology (Terao et al. 2015, Pi et al. 2012, Xiao et al. 2018). BMPER is a critical modulator of the TGF‐β/BMP signaling axis. Prior studies highlight its dual role in cancer progression: BMPER antagonizes BMP4 to inhibit endothelial apoptosis, fostering tumor vascularization (Helbing et al. 2010). In ovarian cancer, BMPER activates MAPK/ERK signaling to enhance epithelial‐mesenchymal transition (EMT) and upregulates matrix metalloproteinases (MMP2/9) (Heinke et al. 2012, Berthelot et al. 2024). This study highlights the critical regulatory role of BMPER in hepatocellular carcinoma (HCC), aligning with previous findings that low BMP‐6 expression promotes HCC progression and poor prognosis. As a BMP regulatory protein, BMPER binds to BMP ligands, inhibiting their activity and modulating osteogenesis and angiogenesis. These molecular‐level interactions suggest that BMPER may act as a tumor suppressor in HCC pathogenesis. Further validation is required to elucidate these context‐dependent mechanisms (He et al. 2014). Herein, to further investigate the mechanism underlying NSUN6 in HCC, bioinformatic analysis was performed, and BMPER was identified as the downstream target regulated by NSUN6. Moreover, further exploration indicated that BMPER expression was downregulated in HCC clinical samples and cell lines, which was associated with poorer survival outcomes. Prior research has concluded that NSUN6 may impact cancer progression by mediating downstream target genes via m5C methylation (Li et al. 2023). Consistent with this observation, our analysis implied that high NSUN6 expression could upregulate BMPER expression in an m5C modification‐dependent manner. It is worthwhile emphasizing that BMPER knockdown substantially attenuated the inhibitory effect of NSUN6 overexpression on HCC cell proliferation and migration in the rescue experiment. These findings further support the hypothesis that BMPER acts as the downstream target of NSUN6 in suppressing HCC development.

Our research elucidated the roles of NSUN6 and its downstream target, BMPER, in HCC, thereby providing valuable insights for clinical applications. Nevertheless, some limitations of this study cannot be overlooked. To begin, the potential of NSUN6 as a prognostic marker across different stages of HCC was not considered, and pathways via which BMPER exerts its functions warrant further exploration.

Collectively, the in vitro and in vivo experiments demonstrated the inhibitory effect of NSUN6 on HCC cell proliferation and migration, as well as on tumor growth. Noteworthily, NSUN6 exerts positive effects by enhancing the stability of BMPER in an m5C‐dependent manner, implying the NSUN6/BMPER axis may serve as a potential therapeutic target for HCC.

## Authors Contribution

**Chunlin Liu**: conceptualization and methodology. **Yumin Wu**: original draft writing. **Ying Wu**: project administration. **Weizhi Luo**: software and validation. **Yuefei Hong**: formal analysis and investigation. **Leichang Jiang**: resources and data curation. **Senrui Wang**: visualization. **Duanming Du**: writing—review & editing and supervision.

## Ethics Statement

All animal experiments were performed in accordance with the ARRIVE Guidelines and approved by the Animal Ethics Committee of Guangzhou Jennio Biotech Co. Ltd. (Approval No. JENNIO‐IACUC‐2023‐A067).

## Conflicts of Interest

The authors declare that they have no conflict of interest.

## Data Availability

All data that support the findings of this study are available from the corresponding authors upon reasonable request.
